# Increasing the translation of mouse models of MERS coronavirus pathogenesis through kinetic hematological analysis

**DOI:** 10.1371/journal.pone.0220126

**Published:** 2019-07-24

**Authors:** Sarah R. Leist, Kara L. Jensen, Ralph S. Baric, Timothy P. Sheahan

**Affiliations:** Department of Epidemiology, University of North Carolina at Chapel Hill, Chapel Hill, North Carolina, United States of America; Deutsches Primatenzentrum GmbH - Leibniz-Institut fur Primatenforschung, GERMANY

## Abstract

Newly emerging viral pathogens pose a constant and unpredictable threat to human and animal health. Coronaviruses (CoVs) have a penchant for sudden emergence, as evidenced by severe acute respiratory syndrome coronavirus (SARS-CoV), Middle East respiratory syndrome CoV (MERS-CoV) and most recently, swine acute diarrhea syndrome coronavirus (SADS-CoV). Small animal models of emerging viral pathogenesis are crucial to better understand the virus and host factors driving disease progression. However, rodent models are often criticized for their limited translatability to humans. The complete blood count is the most ordered clinical test in the United States serving as the cornerstone of clinical medicine and differential diagnosis. We recently generated a mouse model for MERS-CoV pathogenesis through the humanization of the orthologous entry receptor dipeptidyl peptidase 4 (DPP4). To increase the translatability of this model, we validated and established the use of an automated veterinary hematology analyzer (VetScan HM5) at biosafety level 3 for analysis of peripheral blood. MERS-CoV lung titer peaked 2 days post infection concurrent with lymphopenia and neutrophilia in peripheral blood, two phenomena also observed in MERS-CoV infection of humans. The fluctuations in leukocyte populations measured by Vetscan HM5 were corroborated by standard flow cytometry, thus confirming the utility of this approach. Comparing a sublethal and lethal dose of MERS-CoV in mice, analysis of daily blood draws demonstrates a dose dependent modulation of leukocytes. Major leukocyte populations were modulated before weight loss was observed. Importantly, neutrophil counts on 1dpi were predictive of disease severity with a lethal dose of MERS-CoV highlighting the predictive value of hematology in this model. Taken together, the inclusion of hematological measures in mouse models of emerging viral pathogenesis increases their translatability and should elevate the preclinical evaluation of MERS-CoV therapeutics and vaccines to better mirror the complexity of the human condition.

## Introduction

Small animal models of emerging viral pathogenesis are fundamental tools to further our understanding of the molecular and genetic mechanisms driving severe disease outcomes after infection. These models are essential to systematically dissect both viral and host determinants of disease presentation and are key for the critical evaluation of vaccines and antivirals *in vivo*. To maximize the utility, impact and biological relevance of pathogenesis studies or therapeutic evaluation, the measurement of multiple complementary and translatable metrics over time is crucial.

Emerging viral pathogens like Ebola virus, yellow fever virus, Severe Acute Respiratory Syndrome Coronavirus (SARS-CoV) and Middle Eastern Respiratory Syndrome Coronavirus (MERS-CoV) are major threats to global public health [1]. Viral emergence is typically the result of zoonotic virus spill over from animal reservoirs into human populations. Without preexisting immunity, vaccines or therapeutics to newly emerged viral pathogens, sustained human to human transmission coupled with the ease and frequency of human travel could fuel explosive and catastrophic global pandemic disease. In 2012, MERS-CoV was discovered to have emerged from bats through a camel intermediate host in the Middle East, thus far causing 2,428 cases and 838 deaths in 27 countries [2, 3]. Viruses similar to both SARS- and MERS-CoV are currently circulating in bats making the emergence of a SARS- or MERS-like virus in the future a real possibility [4]. Currently there are no approved vaccines or therapies specific for any human CoV.

The complete blood count (CBC) is a routine rapid hematological test that can aid in the diagnosis of blood disorders and infectious disease. In 2016, 42 million CBCs were ordered in the United States, making it the most requested clinical lab test [5]. Moreover, the CBC can be performed without specialized machinery or expensive equipment, providing key clinical information for disease management in patients even in rural or resource limited settings. For example, the CBC described thrombocytopenia and lymphopenia in patients with SARS-CoV [6] and MERS-CoV [7–9] and an increased absolute neutrophil count early during SARS-CoV infection was a strong predictor for intensive care unit (ICU) admission and death [10].

In this study, we characterized the daily variation of different peripheral blood cell populations in a mouse model of MERS-CoV infection and pathogenesis until 4 days post infection (dpi). Peripheral blood was analyzed by an automated veterinary hematology analyzer at biosafety level 3 (BSL3) and by routine flow cytometry techniques. Similar to what was observed in human MERS-CoV patients, we observed significant modulation of leukocytes in MERS-CoV infected mice which was virus dose dependent and measurable prior to notable weight loss. Importantly, neutrophil counts on 1dpi were predictive of disease severity with a lethal dose of MERS-CoV highlighting the predictive value of hematology in this model. These data highlight the predictive value and clinical translatability of the CBC in the study of emerging virus pathogenesis. Therefore, this approach should elevate future studies further dissecting the host factors driving severe MERS-CoV disease and preclinical evaluation of therapeutics and vaccines.

## Materials and methods

### Ethics statement

This study was carried out in strict accordance with the recommendations in the Guide for the Care and Use of Laboratory Animals of the National Institutes of Health. The protocol was approved by the Committee on the Ethics of Animal Experiments of the University of North Carolina at Chapel Hill (Protocol Number: 17–097).

### MERS-CoV pathogenesis model

We previously modified the murine ortholog of the human MERS-CoV receptor, dipeptidyl peptidase 4 (*Dpp4*), in C57BL/6J mice through CRISPR/Cas9 to substitute human residues at positions 288 and 330 (A288L and T330R) thus rendering the mice susceptible to MERS-CoV infection [11]. The resultant mice expressing a humanized DPP4 referred to herein as “288/330^+/+^”were housed in accordance to guidelines set by the Department of Laboratory Animal Medicine at the University of North Carolina at Chapel Hill. Mice were acclimated to the BSL3 environment for one week prior to infection. Ten-week female 288/330^+/+^ mice were randomly assigned to treatment groups, anaesthetized with ketamine and xylazine and infected intranasally with 5.0 x 10^4^ plaque forming units (PFU) mouse adapted MERS-CoV maM35C4 in 50μl of virus collection medium (OptiMEM (Gibco), 3% Fetal Clone 2 serum product (Hyclone) and antibiotic/antimycotic (Gibco) and non-essential amino acids (Gibco)). MERS-CoV maM35C4 is a previously published clonal isolate generated after 35 passages in mice; subsequently plaque purified and clone 4 was expanded two times on Vero CCL81 cells to obtain our working stock grown in virus collection medium [12]. Body weight was measured daily to monitor progression of virus associated morbidity and mortality. Subsets of mice were euthanized via isofluorane overdose and lungs were harvested on days one, two, three and four after infection. The inferior right lobe was frozen at -80˚C and then utilized to measure lung viral load via plaque assay [12]. Results described herein are derived from two independent studies.

To measure fluctuations of blood cells in live mice infected with a lethal or sublethal dose of MERS-CoV, we infected 10 to 11-week old male and female 288/330^+/+^ mice with PBS, 5E+03 or 5E+05 PFU MERS maM35c4 as described above. Body weight was measured daily. On days 1–3 post infection, mice were bled via the submandibular route and blood was analyzed via VetScan HM5. On 4dpi, mice were sacrificed and the inferior right lung lobe was harvested, frozen at -80˚C and then utilized to measure virus lung titer by plaque assay as described above.

### Bronchoalveolar lavage and blood sample collection

Bronchoalveolar lavage (BAL) suspensions were collected immediately following euthanasia by gently injecting 1ml sterile PBS into the mouse lung cavity through the trachea using a catheter attached to a syringe. After 30 seconds, the maximum recoverable volume of PBS (generally 500–800μl) was collected and stored in Eppendorf tubes until prompt VetScan analysis. PBS was not aspirated and re-injected due to the delicate nature of lung tissues following MERS-CoV infection. After lancing the posterior vena cava, peripheral blood was collected in EDTA tubes (Sarstedt) to prevent coagulation. For hematological analysis, 50μl of each blood sample was diluted in 200μl PBS and analyzed via VetScan HM5 (Abaxis) (See below). Remaining BAL and blood samples were saved for flow cytometric analyses (See below).

### VetScan

Peripheral blood collected as noted above was diluted 1:5 in PBS/EDTA and directly analyzed with the VetScan HM5 (Abaxis) fully automated hematology analyzer that was placed within a biological safety cabinet at BSL3. With VetScan HM5, discrimination between cell types is achieved by size. The following parameters were analyzed: lymphocytes (Lym), neutrophils (Neu), monocytes (Mon), basophils (BAS), and eosinophils (EOS), all reported as absolute cell counts as well as percentage of total white blood cells (WBC); red blood cells (RBC), hemoglobin (HGB), hematocrit (HCT), mean corpuscular volume (MCV), mean corpuscular hemoglobin (MCH), mean corpuscular hemoglobin concentration (MCHC), and red blood cell distribution width (RDWc); platelets (PLT), plateletcrit (PCT), mean platelet volume (MPV), and platelet distribution width (PDWc).

### Flow cytometry

For validation of Vetscan reported values, we performed flow cytometry on the identical BAL and whole blood samples after VetScan analysis. Remaining, BAL and whole peripheral blood (100–500μl) samples were stained for flow cytometry. Antibodies used to evaluate the cellular composition of blood were: CD3 (clone 145-2C11; eBioscience), CD19 (clone 6D5), CD45 (clone 30-F11), CD49b (clone DX5), CD115 (clone AFS98), Ly6G (clone 1A8), and NK1.1 (clone PK136; eBioscience). All antibodies were purchased from Biolegend, unless otherwise noted, and were titrated prior to use. Samples were also treated with a Fixable Live/Dead Dye (Invitrogen) to exclude dead cells from downstream analyses. Following antibody staining, erythrocytes were lysed using RBC Lysis Buffer (Biolegend). Samples were acquired using an LSRII cytometer (BD Biosciences) and analyzed using FlowJo software v10.5 (TreeStar). For samples of peripheral blood, samples were run until 50,000+ live, singlet events were acquired, when possible (range 32,000–154,000; mean 99,000 total events). For BAL, samples were acquired until depletion, which resulted in a minimum of 28,000 events (range 28,000–153,000; mean 82,000 events). For analysis, samples were gated to first remove debris, multiplet events, and nonviable cells before positive gates were set using fluorescence minus one staining controls. In addition to expression of CD45, cellular populations were further defined as **i**) “lymphocytes” which included T cells (CD3^+^), B cells (CD19^+^), NK cells (CD3+NK1.1^+^CD49b^+^), and NK T cells (CD3^+^NK1.1^+^CD49b^+^), **ii**) “monocytes” (CD115^+^), and **iii**) “neutrophils” (Ly6G^+^) [13–15]. These cell populations were assigned to reflect to those measured by VetScan HM5.

### Statistical analysis

Data was analyzed using GraphPad Prism (GraphPad Prism Software, San Diego, California). The specific statistical test to determine significance is noted in each figure legend.

## Results

### Peripheral blood leukocyte populations are modulated in MERS-CoV infection

**Fig 1:**
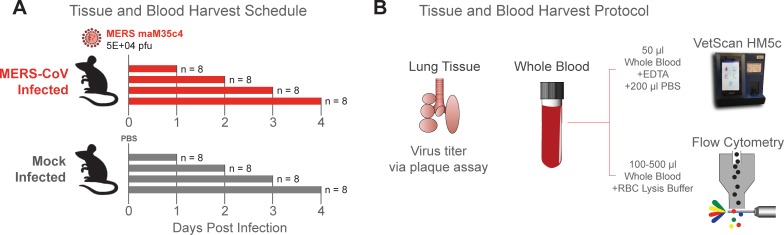
Experimental design. **(A)** Study design outlining infection and sample collection schedule. 10-week-old female 288/330^+/+^ mice were mock infected (PBS, gray) or infected with 5.0 x 10^4^ PFU maM35C4 (red). Per independent experiment, 4 animals/day/group were harvested on 1, 2, 3, and 4dpi (cumulative mice/time/group = 8) for virus titer and hematological analysis. This study was independently repeated once. **(B)** Tissue and blood harvest protocol. Per day, the inferior right lung lobe was harvested, stored at -80˚C until titration by plaque assay of clarified homogenized tissue. Whole blood was harvested, and 50μl was mixed with EDTA and analyzed via VetScan HM5. The remaining whole blood (100–500μl) was mixed with red blood cell lysis buffer, antibody stained and analyzed via multicolor flow cytometry.

**Fig 2:**
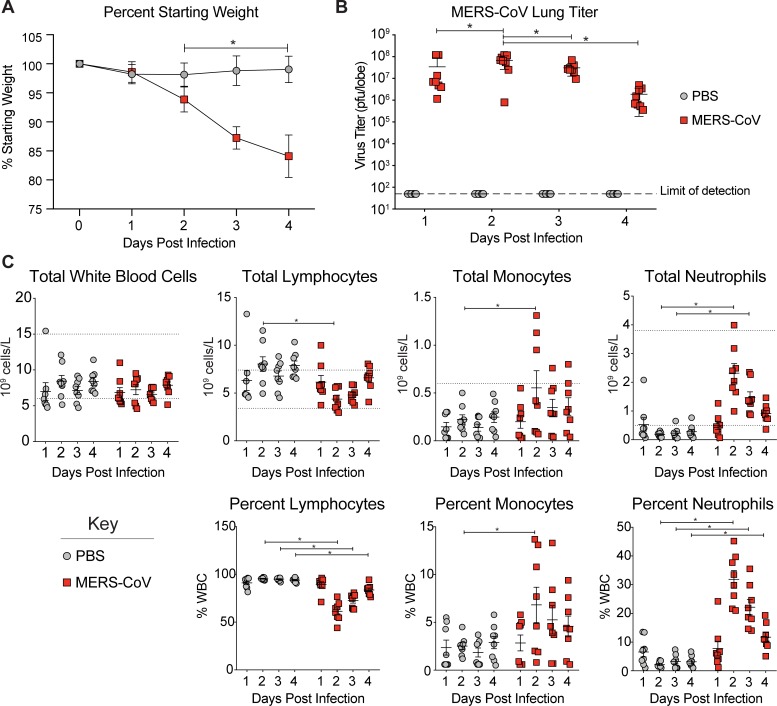
Peripheral leukocyte populations are modulated in MERS-CoV infection. **(A)** Percent starting weight of mock or MERS-CoV infected animals of mice described in Fig 1. The symbols represent the mean per time/group combined from two independent experiments and the error bars represent the standard deviation. Asterisks denote statistical significance as determined by two-way ANOVA with Sidek’s multiple comparison test. **(B)** Virus lung titer via plaque assay of mock or MERS-CoV infected animals combined from two independent experiments. Each symbol represents the titer for a single mouse. The line is at the mean titer and the error bars represent the standard deviation. Asterisks denote statistical significance as determined by two-way ANOVA with Tukey’s multiple comparison test. **(C)** VetScan hematology analysis. Data for total white blood cells (WBCs) as well as the numbers and frequencies of lymphocytes, monocytes and neutrophils are shown. All cell frequencies are expressed as a percentage of total WBCs. Each symbol represents the data from one mouse. The line is at the mean and the error bars represent the standard error of the mean. The dotted lines represent the normal expected range of each cell type according to the manufacturer. Asterisks denote statistical significance as determined by two-way ANOVA with Sidek’s multiple comparison test.

We utilized an established transgenic mouse model of MERS-CoV pathogenesis for these studies where the murine ortholog of the human receptor, dipeptidyl peptidase 4 (DPP4) was humanized at residues 288 and 330 (288/330^+/+^ mice) to facilitate infection and pathogenesis reminiscent to that in humans [12]. We infected 288/330^+/+^ mice with either PBS (mock) or 5E+04 PFU of the mouse adapted MERS-CoV strain maM35C4 and harvested blood and lung tissue for analysis each day after infection for four days (Fig 1A and 1B). Unlike mock infected mice, MERS-CoV infected mice lost significant (P < 0.0001) body weight over time (Fig 2A), which is an expected yet crude marker of MERS-CoV pathogenesis. Similarly, we observed rapid and high titer MERS-CoV replication in lung tissue peaking 2 days post infection (2dpi) and titers were slightly reduced by 4dpi (P < 0.05) (Fig 2B).

To determine if hematological parameters were modulated during MERS-CoV infection, we analyzed peripheral blood from mock or MERS-CoV infected mice at each time point on a VetScan HM5 hematology analyzer (S1 Table). The VetScan HM5 provides a fully automated report of a 22-parameter complete blood count (CBC) from 50μl whole blood, discriminating and quantifying cell numbers based on cell size. The most prevalent white blood cell populations (lymphocytes, neutrophils and monocytes) are enumerated (10^9^ cells/L) and also reported as a percentage of total white blood cells (WBC). Importantly, this machine can be placed in a biosafety cabinet to process infectious samples at biosafety level 3 (BSL3). In mock and MERS-CoV infected animals, levels of total WBCs were not significantly modulated over time with consistent and unvarying cell count and frequency readings obtained at each time point (range P = 0.4 to 0.9) (Fig 2C). While the numbers and frequencies of lymphocytes, monocytes and neutrophils were invariant over time in mock infected mice, these populations were significantly modulated in MERS-CoV infected animals where cell number and frequency were highly correlated (Fig 2C and S1 Fig). By 2dpi, neutrophil (P < 0.0001) and monocyte (P = 0.03) counts and frequencies (neutrophil P < 0.0001, monocyte P = 0.02) were significantly elevated in MERS-CoV infected animals. After 2dpi, the numbers and frequencies of neutrophils remained elevated but slowly waned over the course of our experiment. For monocytes, the variation in cell number and frequency after 2dpi prevented the determination of statistical differences between infected and mock groups (Fig 2C). In infected animals, the numbers of lymphocytes were significantly reduced (P = 0.0004) on 2dpi as compared to mock. Similarly, lymphocyte frequency was reduced in infected animals on 2-4dpi. Other metrics (i.e. RBC counts, hemoglobin, hematocrit, platelets, etc.) measured by VetScan HM5 were not modulated by MERS-CoV infection (S2 Fig). Importantly, the VetScan HM5 phenotypes described above as combined data were reproducible across two independent experiments (S3 Fig). Taken together, systemic immune activation can be detected in peripheral blood following MERS-CoV infection prior to the onset of severe weight loss yet after significant virus replication in the lung.

### Infection mediated modulation of lymphocytes, neutrophils and monocytes measured by Vetscan is corroborated by flow cytometry

**Fig 3:**
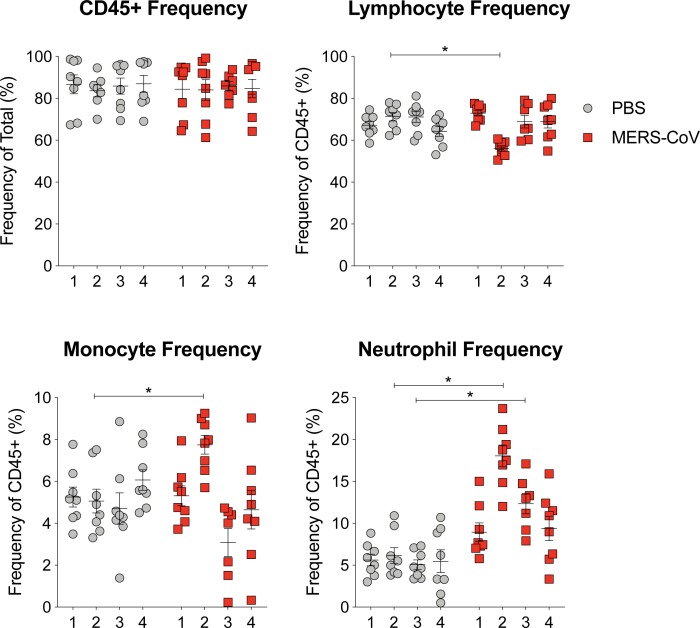
Corroboration of VetScan data by flow cytometry. Whole peripheral blood from mock or MERS-CoV infected mice was stained and analyzed by flow cytometry from two independent experiments and the data was combined. Total white blood cells were identified by cell surface marker CD45. Lymphocyte frequency was generated by combining the frequencies of T, B, NK and NK-T cells. Monocytes and neutrophils were identified by CD115 and Ly6G, respectively. The line is at the mean and the error bars represent the standard error of the mean. Each data point represents the data from one mouse. Asterisks denote statistical significance (P < 0.05) as determined by two-way ANOVA and Sidek’s multiple comparison test.

**Fig 4:**
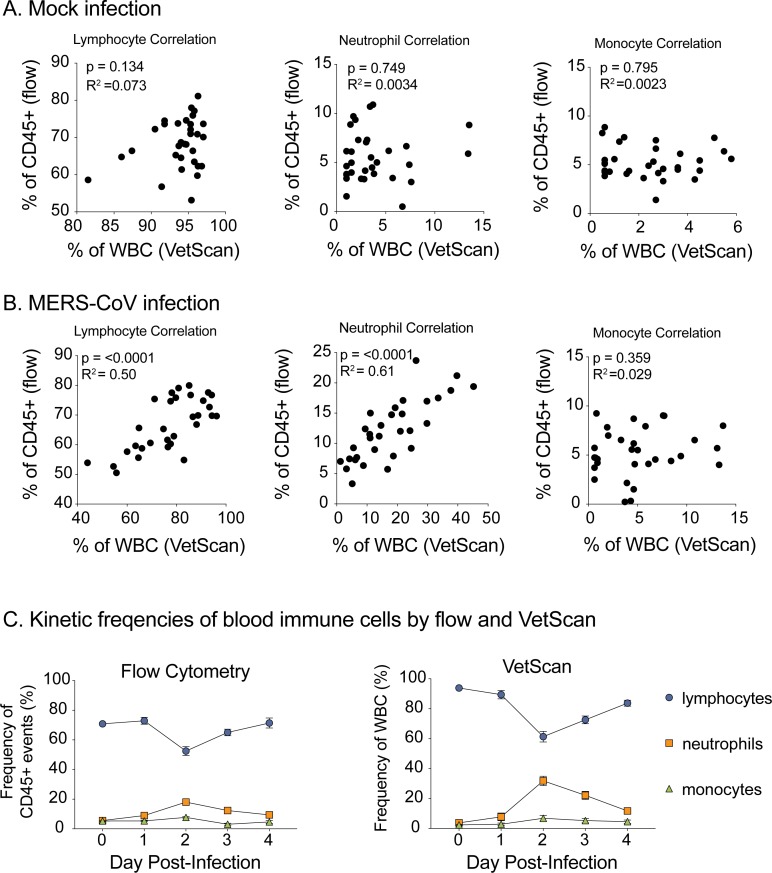
Flow cytometry and VetScan hematological data are highly correlated. **(A)** Pearson Correlation analysis of data from mock infected mice. Lymphocyte (left), neutrophil (center) and monocyte (right) cell frequencies measured by VetScan (x-axes; frequency of WBC) and flow cytometry (y-axes; frequency of CD45^+^ events) from peripheral blood. **(B)** Pearson Correlation analysis data from MERS-CoV infected mice similar to that of **A**. For **A** and **B**, the correlation coefficient (R squared) and p-values obtained are indicated in each plot. **(C)** The kinetic frequencies of blood lymphocytes (circles), neutrophils (squares) and monocytes (triangles) in MERS-CoV infected mice through day 4 post-infection, as measured by flow cytometry (left) and VetScan (right). Time “zero” was generated from mock infected animal data to represent the baseline values. Each symbol represents merged values for 8 animals across two independent experiments; error bars identify median ±SEM.

Since Vetscan HM5 discriminates cell populations based on size, we then sought to validate its accuracy using phenotype staining of paired samples via flow cytometry. While VetScan is designed to enumerate absolute cell numbers from anticoagulated peripheral blood, preparation of blood samples for flow cytometry requires additional processing (e.g. red blood cell lysis) and reports relative cell frequencies based on cell surface marker staining. The cell surface marker, CD45, is a unique marker and is ubiquitously expressed on all white blood cells [16]. Similar to the Vetscan measurement of “total white blood cells”, the frequency of CD45^+^ cells observed in both mock and MERS-CoV infected animals was consistent and invariant over time (Fig 3). Because a single common lymphocyte surface marker does not exist, we combined the independent frequencies of T cell, B cell, NK cell and NK T cell subsets to generate an aggregated “lymphocyte” frequency, to capture the cell types quantified as lymphocytes by Vetscan. Notably, we observed a decrease in the frequency of lymphocytes on 2dpi thus validating the lymphopenia measured by Vetscan HM5. Similarly, monocytes and neutrophil frequencies as measured by flow cytometry in blood were significantly elevated following MERS-CoV infection as compared to mock infected mice mirroring that observed by Vetscan (Fig 3). We then performed Pearson Correlation analysis to compare the frequencies of lymphocytes, neutrophils and monocytes from both flow cytometry and VetScan HM5. Unlike mock infected animals which did not show a correlation among cell frequencies for any cell type (Fig 4A), lymphocyte (P < 0.001, R^2^ = 0.5) and neutrophil (P < 0.001, R^2^ = 0.61) frequency data from MERS-CoV infected mice was highly correlated between flow cytometry and VetScan (Fig 4B). Lastly, the overall trends of cell population frequency modulation as measured by either flow cytometry or VetScan HM5 from the entire time course were remarkably similar (Fig 4C). Thus, two independent techniques generated similar results demonstrating modulation of peripheral leukocyte populations following MERS-CoV infection in mice.

### Numbers of peripheral blood leukocytes on day 1 post infection are predictive of MERS-CoV disease severity

**Fig 5:**
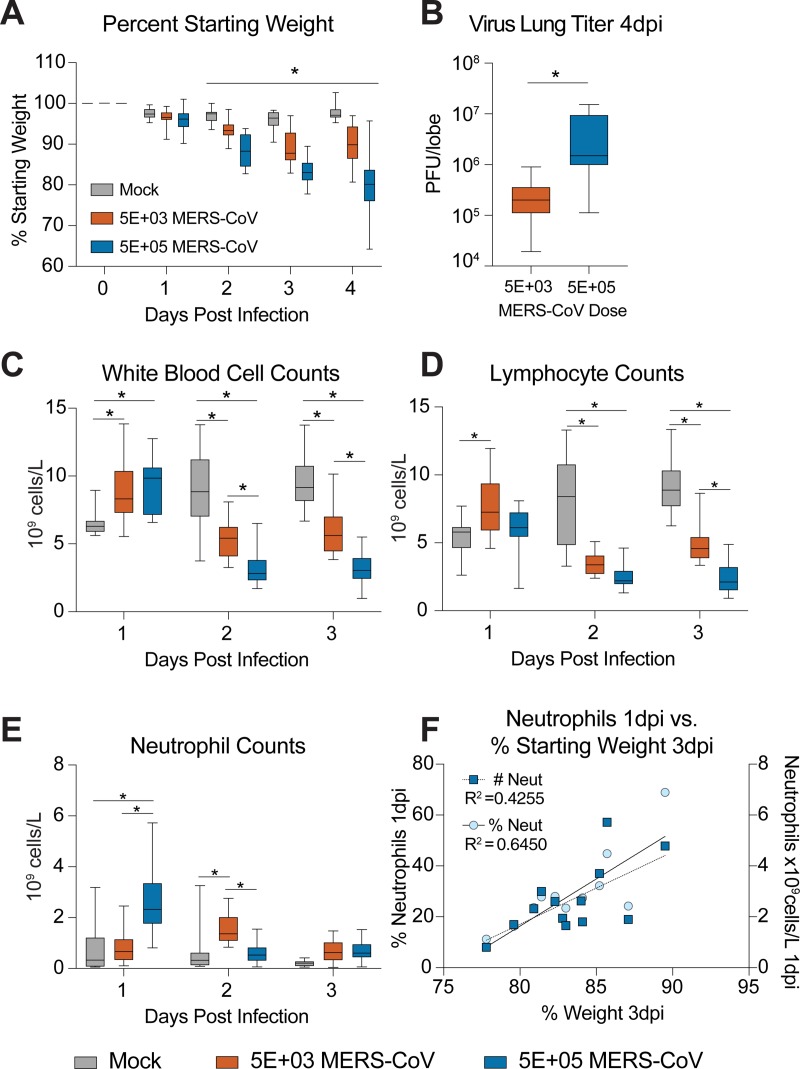
Numbers of peripheral blood leukocytes on day 1 post infection are predictive of MERS-CoV disease severity. **(A)** Percent starting weight of mock infected (N = 10) or those infected with either 5E+03 (N = 13) or 5E+05 (N = 13) PFU MERS-CoV maM35C4. Daily blood samples were obtained by submandibular bleed for each mouse on 1, 2, and 3dpi. **(B)** MERS-CoV virus lung titer on 4dpi via plaque assay. The asterisk indicates a statistically significant difference (P = 0.004) by Mann-Whitney test. **(C)** The numbers of white blood cells in mock or MERS-CoV infected mice described in **A**. **(D)** The numbers of lymphocyte cells in mock or MERS-CoV infected mice described in **A**. **(E)** The numbers of neutrophil cells in mock or MERS-CoV infected mice described in **A**. For the box and whisker plots in **A**, **B**, **C**, **D** and **E**, the line is at the median, the box extends from the 25^***th***^ to 75^***th***^ percentile and the whiskers encompass the range. For **A**, **C**, **D** and **E**, the asterisk indicates statistically different values by Two-Way ANOVA with Sidek’s multiple comparison test. **(F)** Weight loss on 3dpi in high dose MERS-CoV (5E+05 PFU) infected mice is correlated with % neutrophils in peripheral blood on 1dpi. Linear regression was performed to determine correlation. The goodness of fit R^***2***^ value is shown in the plot.

To determine if the numbers of leukocytes in peripheral blood early in MERS-CoV infection are predictive of disease severity, we infected 10–11 week old male and female 288/330^+/+^ mice with either PBS (mock) or 5E+03 or 5E+05 PFU MERS maM35C4. These doses have previously been shown to cause sub-lethal (5E+03) and lethal (5E+05) disease in our model [12]. Since our above analysis had been performed on blood from terminal cardiac puncture, in this study we bled live mock and MERS-CoV infected mice via submandibular bleed to obtain longitudinal CBC data per mouse. In addition, the studies described in previous figures were all performed with a dose of MERS-CoV in between (i.e. 5E+04 PFU) those described in Fig 5. Similar to previously reported data [12], we observed MERS-CoV dose dependent weight loss with sublethal and lethal doses of MERS-CoV where all groups were significantly different from each other beginning 2dpi (Fig 5A, P value range P = 0.0461 to P = <0.0001). Virus lung titer was also different among infected groups on 4dpi (P = 0.004) (Fig 5B). While we did not see significant differences in total WBC counts among mock and MERS-CoV infected (5E+04 PFU) mice in Fig 2C, we observed virus dose dependent differences in this metric when comparing mock, sublethal and lethal doses of MERS-CoV (Fig 5C). Although there was an increase in WBCs with both doses of virus on 1dpi as compared to mock infection, on 2 and 3dpi leukopenia was observed (Fig 5C). CBC derived from cardiac puncture and submandibular bleed of mice have been shown by others to be equivalent[17]. Thus, the differences in WBC trends in Figs 2 and 5 are not likely because of bleeding route and are more likely related to the differences in virus dosage and/or slight differences in the numbers of measured cells (i.e. lymphocytes, monocytes, neutrophils, etc.) which revealed (Fig 5) or obscured (Fig 2) trends in the WBC metric. Similar to Fig 2C, MERS-CoV infection caused significant lymphopenia on 2 and 3dpi with both sublethal and lethal doses virus (Fig 5D). In addition, the numbers of neutrophils were elevated on 1dpi with the lethal dose but this increase was kinetically delayed in the sublethal dose to 2dpi (Fig 5E). Thus, neutrophil elevation kinetics in peripheral blood was differentially modulated with virus dose (Figs 2C and 5E). Importantly, in mice infected with a lethal dose of MERS-CoV, there was a positive correlation between weight loss on 3dpi and the percentage and total numbers of neutrophils in blood on 1dpi (Fig 5F). These data demonstrate that the levels of peripheral blood leukocytes correlate with MERS-CoV disease in a dose dependent manner. More specifically, neutrophil numbers on 1dpi in peripheral blood of mice infected with a lethal dose of MERS-CoV was predictive of disease severity.

### The potential application of VetScan technology to evaluate bronchoalveolar lavage fluid

After establishing the utility of the VetScan HM5 to perform routine hematology on MERS-CoV infected mice, we then evaluated the potential for VetScan to characterize leukocytes in extra-hematological samples more directly relevant to respiratory infection, such as bronchoalveolar lavage (BAL). The VetScan instrument requires a minimum cell density to generate a reading. We found that BAL samples generated measurable results using VetScan HM5, but only under certain experimental conditions. Although we were able to characterize BAL isolated cells by flow cytometry (manuscript in preparation) in all animals regardless of infection status, Vetscan only intermittently provided reliable readings in MERS-CoV infected animals at late times post infection (3dpi and 4dpi) (S2 Table). Thus, most BAL samples from mock and MERS-CoV infected did not exceed the threshold required to generate reliable enumeration by VetScan HM5. We attempted to overcome this issue through concentration of the BAL samples but were still unable to generate reliable reads for both, mock and MERS-CoV infected animals. Thus, without further optimization of enumerating immune cells in BAL via VetScan HM5, flow cytometry remains the ideal application for evaluating immune population dynamics in these kinds of samples.

## Discussion

Animal models of emerging viral diseases are essential to provide insight into the virus and host determinants of pathogenesis and facilitate the evaluation of experimental therapeutics. To maximize the translation of these models, methods of non-invasive longitudinal data collection for multiple metrics on individual animals reminiscent of those collected on human patients are needed. Moreover, models often rely on animal weight loss, a relatively non-specific marker, to chronicle disease which may overlook critical markers of disease progression and severity. Due to the ubiquity of the CBC in the diagnosis and triage of emerging viral diseases, we sought to establish this technique at BSL3 to better understand the modulation of blood cells in a mouse model of MERS-CoV pathogenesis, thus increasing its translation to the human condition. In kinetic studies, we compared data generated by an automated veterinary hematology analyzer, VetScan HM5, to those from standard flow cytometry. Unlike flow cytometry, which reports relative cell frequencies by defined antigen staining, the VetScan HM5 identifies and enumerates the most prominent blood cell types based on size. Furthermore, the VetScan measures several other parameters in addition to cell frequencies within a single read (e.g. platelets, hematocrit and hemoglobin, etc.). In our MERS-CoV mouse model, we determined that VetScan HM5 could accurately measure fluctuations of major immune cell populations (i.e. lymphocytes, monocytes and neutrophils), which mirrored results generated by flow cytometry. After MERS-CoV infection, we observed lymphopenia, neutrophilia and elevated monocytes in peripheral blood by two different methodologies. Importantly, we demonstrated that the disease severity could be differentiated by CBC performed daily in a longitudinal study of mice infected with either a sublethal or lethal dose of MERS-CoV. While flow cytometry is the gold standard used to characterize immune cell populations, this technique requires technical expertise, expensive reagents and equipment, and often significant sample processing time especially for those working at BSL3 where biosafety procedures complicate this already complex process. The VetScan HM5 offers a simple and rapid alternative to flow cytometry when aiming to understand general responses in peripheral blood in emerging models of viral pathogenesis, especially those studied in BSL3 environment. Thus, the data from VetScan HM5 could serve to inform experimental design (i.e. choose timepoints) and simplify more rigorous immune cell phenotyping by flow cytometry for BSL3 pathogens.

Small animal models of emerging viral pathogenesis are fundamental tools that further our understanding of human infectious diseases. These models are essential to systematically dissect both viral and host determinants of disease and are key for the critical evaluation of vaccines and antivirals. To maximize the utility, impact and biological relevance of pathogenesis studies or therapeutic evaluation, the measurement of multiple complementary and translatable metrics is crucial. Recently, several transgenic mouse models have been generated on the C57BL/6 background that facilitate the study of MERS-CoV pathogenesis through the modification of the murine ortholog of the human MERS-CoV receptor, dipeptidyl peptidase 4 (DPP4). Although the genetic approaches vary from complete replacement of murine DPP4 with human DPP4 [18], replacement of exons 10–12 in mouse *Dpp4* with those of human DPP4 [19], or single amino acid changes at residues 288 and 330 to “humanize” murine DPP4 [11], these models have offered complementary insights into the viral and host factors that contribute to severe lung disease after MERS-CoV infection. Only one of these studies performed CBC [19] but this was performed manually on blood smears to obtain percentages of lymphocytes, monocytes and neutrophils and only on 3 and 4 dpi. Nevertheless, the data that Li *et*. *al* reported with mouse adapted MERS-CoV infection of their transgenic mice are concordant with those reported herein with overall reductions lymphocyte percentages and increases in monocytes and neutrophils. Interestingly, using blood smears, Roberts *et*. *al* reported lymphopenia and neutrophilia following infection of BALB/c mice with mouse adapted SARS-CoV strain MA15 [20]. Thus, VetScan has potential utility in characterizing the biology of multiple models of emerging CoV disease.

Viral infections often cause an alteration of peripheral blood cell population frequencies during their disease progression. In combination with other tools commonly utilized to aid in diagnosis and treatment (i.e. patient history, physical exam, vital signs, blood chemistry, etc.), a thorough understanding of the kinetics of blood cell population modulation can help in determining stage of the infection, clinical severity and inform possible therapeutic avenues. For example, even before SARS-CoV was identified as the etiological agent of the SARS-CoV outbreak in 2003, clinicians in Hong Kong noted elevated neutrophil counts upon admission of patients with SARS-CoV and a continued decline in lymphocyte counts during the first week of hospitalization [10]. Additionally, advanced age, elevated serum lactate dehydrogenase, and elevated neutrophil counts at admission were all predictors of adverse outcomes. Many of the clinical manifestations of SARS-CoV infection, such as lymphopenia, elevated neutrophils and increase serum LDH, were also observed in MERS-CoV patients [7–9]. Thus, the observation reported here of decreased numbers of lymphocytes and increased neutrophils in the peripheral blood of MERS-CoV infected mice faithfully recapitulates those manifestations in infected humans. In fact, in our comparative longitudinal study comparing sublethal and lethal doses of MERS-CoV in Fig 5, we found that neutrophil numbers in the peripheral blood on 1dpi correlated with degree of body weight loss 3dpi in the lethal dose group. Thus, with a lethal dose of MERS-CoV, neutrophil numbers prior to the onset of significant weight loss were predictive of disease severity as measured by weight loss on a per mouse basis. Therefore, neutrophils in peripheral blood prior to the onset of severe clinical disease may serve as a predictive biomarker of severe MERS-CoV disease in mice, just as elevated neutrophils at the time of SARS-CoV patient admission predicted adverse outcomes in humans.

The studies described herein highlight the predictive value and clinical translatability of the CBC in the study of emerging virus pathogenesis. This technique when coupled with whole body plethysmography to measure pulmonary function [21], Bio-Plex to measure cytokines and chemokine levels in the lung, flow cytometry to immunophenotype inflammatory cells in the lung, viral load and histopathology will yield a more comprehensive view of emerging viral pathogenesis and increase the biological relevance of mouse models of emerging CoV disease. Therefore, the inclusion of the CBC should elevate, increase the biological relevance and translation of future studies of emerging virus pathogenesis as well as the preclinical evaluation of therapeutics and vaccines.

## Supporting information

S1 Fig High correlation of total lymphocyte, neutrophil and monocyte cell counts and cell frequencies in peripheral blood measured by Vetscan. **(A)** The correlation of lymphocyte (left), neutrophil (center) and monocyte (right) cell counts (cells/L) with cell frequencies (% of WBC) reported from VetScan reads on peripheral blood in mock infected mice. **(B)** The correlation of lymphocyte (left), neutrophil (center) and monocyte (right) cell counts (cells/L) with cell frequencies (% of WBC) reported from VetScan reads on peripheral blood in MERS-CoV-infected mice. Pearson R squared and p-values are indicated on each plot.
(EPS)

S2 Fig Red blood cells, hemoglobin, hematocrit and platelets are not modulated after MERS-CoV infection. Red blood cells, hemoglobin, hematocrit and platelets as determined by VetScan HM5. Each symbol represents the data from one mouse. The data are combined from two independent studies. Each time point per group is created from 8 individual mice. The line is at the mean and the error bars represent the standard error of the mean. Differences were not statistically significant by Two-way ANOVA with Tukey’s multiple comparison test.
(EPS)

S3 Fig VetScan data reproducibility between independent experiments. **(A)** The WBC, lymphocyte, neutrophil and monocyte counts and frequencies of WBC are shown for mock infected animals processed during two independent experiments (light and dark grey differentiate experiment 1 and 2). **(B)** The WBC, lymphocyte, neutrophil and monocyte counts and frequencies for animals experimentally infected with MERS-CoV. Error bars indicate the median ±SEM.
(EPS)

S1 Table Mock or MERS-CoV infected mouse peripheral blood samples collected for VetScan and flow cytometry analysis across experiments 1 and 2. Except for one animal at one timepoint, blood samples from all animals in both experiments and both treatment groups had readings by VetScan HM5 and flow cytometry.
(DOCX)

S2 Table Mock or MERS-CoV infected mouse bronchoalveolar lavage (BAL) samples collected for VetScan and flow cytometry analysis across experiments 1 and 2. BAL samples were recovered from all animals and run by VetScan and flow cytometry to evaluate the frequencies of immune cell subsets. However, most VetScan readings from experiment 1 for BAL samples were too dilute to generate measurements; just one infected animal on days 3 and 4 post infection contained sufficient cellular density to generate a VetScan reading within range. During experiment 2, BAL samples were concentrated in an attempt to improve VetScan read efficiency. Concentrating samples did produce a greater proportion of usable reads, particularly in MERS-CoV-infected animals, but reads from uninfected and infected animals day 1 post-infection were still outside of VetScan range. Flow cytometry analysis of BAL samples from experiments 1 and 2 were possible, demonstrating that the BAL collection method did result in immune cell recovery.
(DOCX)
