# Supplementary material for: Increasing the translation of mouse models of MERS coronavirus pathogenesis through kinetic hematological analysis
Source: PLoS One. 2019 Jul 24;14(7):e0220126. doi: 10.1371/journal.pone.0220126 (PMC6655769; doi:10.1371/journal.pone.0220126)
Supplement: S1 Table — Except for one animal at one timepoint, blood samples from all animals in both experiments and both treatment groups had readings by VetScan HM5 and flow cytometry. (DOCX) [file pone.0220126.s004.docx]

|  |  |  |  | **EXPERIMENT 1** | |  | **EXPERIMENT 2** | |
| --- | --- | --- | --- | --- | --- | --- | --- | --- |
|  |  |  |  | **+ PBS** | **+ MERS** |  | **+ PBS** | **+ MERS** |
| **BLOOD** | day 1 | VetScan |  | 4 | 4 |  | 4 | 4 |
|  |  | Flow |  | 4 | 4 |  | 4 | 4 |
|  | day 2 | VetScan |  | 4 | 4 |  | 4 | 4 |
|  |  | Flow |  | 4 | 4 |  | 4 | 4 |
|  | day 3 | VetScan |  | 4 | 4 |  | 4 | 4 |
|  |  | Flow |  | 4 | 3 |  | 4 | 4 |
|  | day 4 | VetScan |  | 4 | 4 |  | 4 | 4 |
|  |  | Flow |  | 4 | 4 |  | 4 | 4 |
