# Supplementary material for: Increasing the translation of mouse models of MERS coronavirus pathogenesis through kinetic hematological analysis
Source: PLoS One. 2019 Jul 24;14(7):e0220126. doi: 10.1371/journal.pone.0220126 (PMC6655769; doi:10.1371/journal.pone.0220126)
Supplement: S2 Table — BAL samples were recovered from all animals and run by VetScan and flow cytometry to evaluate the frequencies of immune cell subsets. However, most VetScan readings from experiment 1 for BAL samples were too dilute to generate measurements; just one infected animal on days 3 and 4 post infection contained sufficient cellular density to generate a VetScan reading within range. During experiment 2, BAL samples were concentrated in an attempt to improve VetScan read efficiency. Concentrating samples did produce a greater proportion of usable reads, particularly in MERS-CoV-infected animals, but reads from uninfected and infected animals day 1 post-infection were still outside of VetScan range. Flow cytometry analysis of BAL samples from experiments 1 and 2 were possible, demonstrating that the BAL collection method did result in immune cell recovery. (DOCX) [file pone.0220126.s005.docx]

|  |  |  |  | **EXPERIMENT 1** | |  | **EXPERIMENT 2** | |
| --- | --- | --- | --- | --- | --- | --- | --- | --- |
|  |  |  |  | **+ PBS** | **+ MERS** |  | **+ PBS** | **+ MERS** |
| **BAL** | day 1 | VetScan |  | 0 | 0 |  | 0 | 0 |
|  |  | Flow |  | 4 | 4 |  | 4 | 4 |
|  | day 2 | VetScan |  | 0 | 0 |  | 1 | 3 |
|  |  | Flow |  | 4 | 4 |  | 4 | 4 |
|  | day 3 | VetScan |  | 0 | 1 |  | 0 | 4 |
|  |  | Flow |  | 4 | 4 |  | 4 | 4 |
|  | day 4 | VetScan |  | 0 | 1 |  | 0 | 4 |
|  |  | Flow |  | 4 | 4 |  | 4 | 4 |
